# Lived experience of healthcare providers amidst war and siege: a phenomenological study of Ayder Comprehensive Specialized Hospital of Tigray, Northern Ethiopia

**DOI:** 10.1186/s12913-024-10655-3

**Published:** 2024-03-06

**Authors:** Awol Yemane Legesse, Znabu Hadush, Hale Teka, Ephrem Berhe, Bisrat Tesfay Abera, Fasika Amdeselassie, Hiluf Ebuy Abraha, Daniel Gebre, Alessandra N Bazzano

**Affiliations:** 1 College of Health Sciences, Department of Obstetrics and Gynecology, Mekelle University, Tigray, Ethiopia; 2 College of Health Sciences, School of Public Health, Mekelle University, Tigray, Ethiopia; 3 College of Health Sciences, Department of Internal Medicine, Mekelle University, Tigray, Ethiopia; 4 College of Health Sciences, Department of Surgery, Mekelle University, Tigray, Ethiopia; 5 Ayder Comprehensive Specialized Hospital, Mekelle University, Quality Office, Tigray, Ethiopia; 6 Ayder Comprehensive Specialized Hospital, Mekelle University, Labor ward, Tigray, Ethiopia; 7 Tulane University School of Public Health and Tropical Medicine, New Orleans, LA USA; 8 University of South Carolina, Arnold School of Public Health, Columbia, SC USA

**Keywords:** Tigray war, Ethiopia, Siege, Health professionals, Lived experience

## Abstract

**Background:**

Most wars are fought in poor countries and result in significant proportions of disabilities and mortalities. The consequences of wars and political instability on health workers and access to healthcare remain under-studied. This study aimed to explore the lived experience of healthcare providers amidst war and siege, in a teaching hospital in northern Ethiopia.

**Methods:**

The study was conducted between February 2022 to March 2022. A qualitative phenomenological study was conducted between February to March 2022 with 20 healthcare providers working in Ayder Comprehensive and Specialized Hospital (ACSH), Tigray, Ethiopia, during the Tigray War. The study employed in-depth interviews.

**Results:**

The main themes identified included the consequences of the siege on health service delivery at ACSH, personal survival threats posed by the siege, immediate health consequences of the siege among care providers, and consequences of the siege on the motivation and energy of health professionals.

**Conclusions:**

Health workers are exposed to a range of direct and indirect impacts of war, emphasizing the need to amend the conditions in which they live and work.

**Supplementary Information:**

The online version contains supplementary material available at 10.1186/s12913-024-10655-3.

## Background

Low-income country populations are beset by challenges to health and safety, including levels of conflict and war not typically present in higher-income countries, resulting in significant disability and mortality [1, 2]. Many of these conflicts involve “regimes at war with sectors of their society” – generally targeting an ethnic group within the country [3]. The mechanisms of domination and oppression in these countries are through deliberate destruction of the social fabric, economic control, and repression of cultural life, as well as atrocities committed such as extrajudicial killings, forced disappearances, and using rape as a weapon of war [4–7].

Often, when war is considered, the foremost concern is the number of deaths and injuries [5, 8, 9]. However, there are many other consequences of war and conflict that warrant careful consideration. Among such consequences, the threats to health workers and dire limitations on access to healthcare that often result from war and long-term political instability cannot be over-emphasized [10–14]. In a conflict setting, healthcare institutions are often targeted by warring parties [11, 12, 15]. In 2019, at least 1,200 attacks on healthcare workers and health workers occurred in 20 countries [16, 17]. Targeting healthcare institutions by troops in Syria, Iraq, Yemen, and the war in Tigray, Ethiopia has left 70% of healthcare institutions dysfunctional [18–23]. Despite carrying out the crucial task of caring for the sick, healthcare workers have increasingly become targets during war [24]. Simultaneous with the fear of being a victim of collateral damage in violence is the fear on the part of healthcare workers that they will be deliberately targeted. To preserve their own lives, healthcare workers flee conflict-affected areas. An obvious but sad ramification is that once the healthcare workers leave a war-torn area, they can no longer administer the vital care that is badly needed. A study conducted by the International Committee of the Red Cross reported that violence against health workers in war-torn areas is ‘one of the most crucial, yet overlooked humanitarian issues of today’ [25]. It is over a century and a half since the first Geneva Convention, an international prohibition of “attacking the sick, the wounded, assaulting those who offer them healthcare” [26]. However, as attacks against healthcare institutions and providers continue to escalate, this convention remains neither observed nor enforced [27]. 

While health professionals are bound by their professional ethics and the Geneva Conventions; [26] the legal, moral, and ethical doctrines under which they operate can be seriously defied. Unlike in peaceful times, several considerations must be taken into account during wartime such as the resources available, the security situation, and the capacity to deal with other medical priorities. Treatment of sick and injured people may not be technically or physically possible and there may be no option to transfer patient care to other facilities. Health professionals in a war zone are deeply affected by their environment. Not only can they risk their own lives, but the effects of working through constant fatigue and dealing with horrific injuries contribute to secondary trauma and compassion fatigue [28]. Although several studies have examined the conditions of healthcare professionals during times of war, there is a lack of information on their experiences during prolonged periods of siege [11–13]. Therefore, this study aims to delve into the experiences of healthcare providers during such situations, seeking to understand their perspectives and navigate the complex mix of emotions, challenges, and triumphs that they encounter in their daily struggles. By shedding light on their experiences, the study aims to contribute to a deeper understanding of healthcare costs during wartime and the resilience of healthcare providers.

## Methods

In this study, we aim to explore the work and living conditions of healthcare professionals at Ayder Comprehensive Specialized Hospital (ACSH), a teaching hospital located in Tigray, northern Ethiopia, during times of war and siege.

### Study design

A qualitative phenomenological (Merleau-Ponty’s idea of perception) study design was employed to explore the lived experiences of healthcare workers during the siege and conflict in Tigray Region, Ethiopia from February 2022 to March 2022.

### Study setting

The war in Tigray broke out in November 2020 and Ethiopian Federal troops took control of the capital of the region, Mekelle, a month later. Seven months following this, federal troops were forced out of most parts of Tigray by the opposition forces. Subsequently, all ties were severed and the Federal Government cut off all resources to the Region. With this, the Tigray region was besieged. During this time there was an active war on almost all fronts surrounding the region, and there were continuous drone and aerial bombings in multiple areas of Tigray, including the capital Mekelle.

### Population

Healthcare providers specified in the categories of Senior Practitioners, Residents, Interns, Nurses, and Midwives who were working at Ayder Comprehensive Specialized Hospital (ACSH) during the time of the data collection were eligible for the study. Based on the criteria specified before data collection, care providers who were recruited essentially were the actual study population of the present study.

### Sample size and sampling procedure

Healthcare providers were purposively selected using the following representativeness criteria: (1) profession/job title, (2) gender, (3) marital status, and family size, (4) work experience, and (5) position in their health service unit. The investigators conducted an expert discussion to identify and set relevant criteria for the purposive participant selection. The criteria were assumed to identify potential participants with rich information from their lived experiences regarding the impact of the siege in Tigray on the life and work conditions of healthcare providers in the hospital, with variation in perspectives. Consequently, the initial sample size was 20 healthcare providers. Four participants per professional group (senior physicians, residents, interns, nurses, and midwives) were selected. To ensure gender mix, half of the participants were female healthcare providers. To enhance the varied composition of the potential participants, seven out of the twenty participants were single in marital status and eight were individuals who possessed a position at the time of the siege. In addition, work experience was considered for additional variation. The above criteria were used to ensure a diverse and representative sample of health professionals who have experienced war and siege. Position is important to understand the experiences of different levels of healthcare workers, while gender and marital status influence how individuals experience and cope with the stressors of war and siege. Work experience is also relevant to understanding how individuals’ prior experiences in healthcare may shape their responses to the current situation. Overall, these variables were chosen to provide a comprehensive understanding of the lived experiences of health professionals in Tigray during periods of war and siege.

The maximum sample size was determined to be 20 care providers using the principle of saturation of information, phenomenological grounding, and considering resource limitations. Table 1 summarizes the characteristics of the study participants.

**Table 1:** Characteristics of the lived experience of healthcare providers amidst war and siege study participants in ACSH, Tigray, February-March, 2022

Participant Groups	Gender		Marital Status		Experience in ACSH		Position in Service unit*	
	Male	Female	Married	Single	< 5 years	5years & above	Yes	No
Seniors	2	2	3	1	0	4	2	2
Residents	2	2	3	1	2	2	0	4
Interns	2	2	2	2	2	2	0	4
Nurses	2	2	2	2	2	2	2	2
Midwives	2	2	3	1	2	2	2	2
**Total**	**10**	**10**	**13**	**7**	**8**	**12**	**6**	**14**

* Leadership role

### Data collection tools and procedure

The investigators, who are located within different disciplinary areas, developed a semi-structured interview guide with open questions to capture the variety and depth of the lived experiences and working conditions of healthcare providers during the siege in Tigray. Investigators conducted peer debriefs regarding the ongoing data collection process for reflexivity and consensus on approaches, and the interview guide was subject to modification after each interview to incorporate the emerging salient ideas in the forthcoming interview.

Five trained investigators collected audio-recorded data, in Tigrigna, the local language of the region, transcribed it word by word, and translated it into English. Before the interview, participants were asked for a convenient time and place for the interview to secure their privacy. Investigators conducted the interviews in a private place where recording was possible with minimal noise. The interview lasted for 45–120 min and was audiotaped using recorders. The interviewers also used detailed field notes to capture experiences as well as non-verbal expressions. Information from field notes for each interview was annotated to each transcript to be considered in the data analysis process. To improve data rigor and richness, the investigators asked follow-up questions for opinions and answers that needed elaboration, confirmation, or clarification. As investigators were also from the settings where the siege was imposed, they ‘bracketed’ their own opinions, experiences, and preconceptions per the phenomenological paradigm, to reduce the introduction of potential bias during data collection, transcription, coding, and analyses.

### Trustworthiness

To ensure confirmability, the investigators utilized multiple sources of data and triangulated findings. To ensure credibility, the investigators were utilizing member checking, peer debriefing, and prolonged engagement with participants. During periods of independent coding and peer debriefing, all investigators minimized the risk of subjectivity bias. To improve the quality of the findings, investigators withheld (bracketing) their expectations and previous knowledge while interviewing participants, generating coding, and grouping codes into categories and themes. Finally, member checking was conducted with five participants, one participant from each category of health professionals to improve the trustworthiness of the findings.

### Data analysis

#### Identification of specific codes

##### Data collection and transcription

The investigators conducted in-depth interviews with 20 health professionals who had experienced war and siege in Tigray, Ethiopia. The interviews were transcribed verbatim (in the local language) and translated into English. Each translated interview was saved as an independent file in MS Word file and imported into Atlas.ti qualitative data analysis software version 7.5 (ATLAS.ti Scientific Software Development GmbH, Berlin, 2015) for coding and analysis.

##### Initial coding process

Two investigators independently read through the transcripts to identify initial codes related to the participants’ experiences. Where there was variation in coding the texts, they discussed and invited a third investigator for consensus in coding. In addition, field notes and investigator memos were also linked to respective files in the software to assist in analysis.

##### Codebook development

The investigators developed a codebook that included codes such as “fear,” “trauma,” “anxiety,” “dissatisfaction,”

#### Categorization of codes

##### Grouping similar codes into categories

The investigators grouped similar codes together to create categories such as “social interaction,” “Coping Strategies,” and “motivation and energy.”

##### Creating subcategories within categories

Within the category of “social interaction” subcategories were created for different types of interaction such as loneliness, isolation, and aggressiveness.

#### Refinement of codes

##### Reviewing and revising codes for clarity and consistency

The investigators reviewed the codes to ensure they were clear, concise, and consistent with the data.

##### Consolidating redundant or overlapping codes

The investigators consolidated redundant or overlapping codes to reduce redundancy in the analysis.

#### Theme development

##### Identifying key themes across categories and subcategories

The investigators identified key themes such as " Personal survival threats posed by the siege,” “Consequences of the siege on health service delivery at ACSH,” and " Consequence of the siege on motivation and energy of health professionals.”

Analyzing relationships between themes: The investigators analyzed the relationships between themes to gain a deeper understanding of the participants’ experiences. For example, they found that personal survival threats were detrimental to the decline of motivation and energy of health professionals and subsequently had a negative impact on the service provision.

## Results

**Fig. 1:**
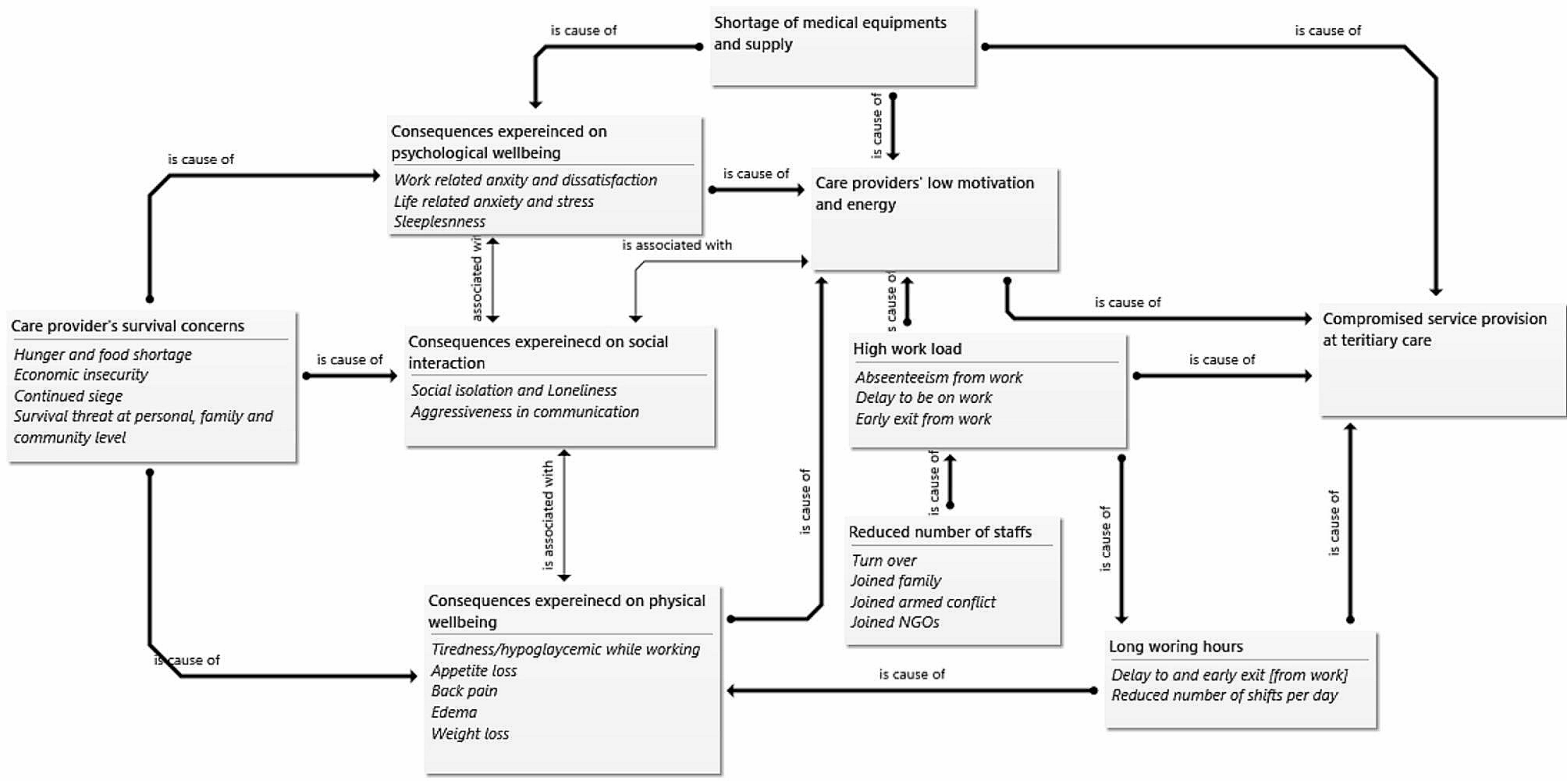
Diagrammatic presentation of the main consequences of Tigray siege on the work and life conditions of healthcare providers in the Ayder Comprehensive Specialized Hospital, Tigray Region, Northern Ethiopia

The current study revealed the Tigray siege significantly affected the life and work conditions of care providers in ACSH. The immediate consequences of the siege in Tigray were evident to affect four main components of ACSH; it affected service delivery; it posed a survival threat for the care providers; it impacted the care providers’ well-being; and the morale and motivation of the healthcare workers to provide care also declined. The themes identified are presented in Table 2 below and additional main findings of the study are visually represented in the figure below (Fig. 1). Themes and sub-themes are presented in detail in subsequent paragraphs.

**Table 2:** Main themes of the lived experience of healthcare providers amidst war and siege study participants in ACSH, Tigray, February-March, 2022

Theme	Description
**1**	Consequences of the siege on health service delivery at ACSH
	Compromised service provision at ASCH Shortage of medical equipment and supply
**2**	Personal survival threats posed by the siege
	Hunger and food shortage Economic insecurity
**3**	Immediate health consequences of the siege among care providers
	Consequences experienced in physical well being Consequences experienced in psychological well being Consequences experienced in social interaction
**4**	Consequence of the siege on the motivation and energy of health professionals
	Health professionals’ low motivation and energy

### Theme 1: consequences of the siege on health service delivery at ACSH

This theme related to the quality of care that the hospital could provide being seriously compromised. Patients who could survive under normal conditions have died due to a lack of essential medications, including antibiotics, drugs for chronic medical illnesses, drugs for labor and delivery, lack of personal protective equipment, anesthesia, and supplies, including oxygen and equipment needed for the operating theatre. Much of the care that was provided deviated far from expected standards. The imposed siege had also led to high staff attrition and subsequent high workload among those on duty.

### Absence of drugs and medical supplies

According to the participants, though the general decrease in the availability of drugs and medical supplies started early in the aftermath of war, intermittent provision of supplies continued until late June of 2021. Following the siege, however, the provision of even the most basic services become impossible. Consequently, the absence of normal saline/IV fluids, glucose, antibiotics, drugs for chronic medical illness, and supplies to assist labor and delivery such as oxytocin and misoprostol, anesthetics, oxygen supply, blood, and basic equipment needed for Operating Theater (OR) and dialysis were the most frequently mentioned. The participants also revealed the scarcity of basic laboratory tests and the lack of maintenance of imaging machines, which have seriously challenged the provision of services. The following quote supports these reflections.

The lack of medical equipment has become more problematic for us than any other constraints. We are witnessing the death of patients due to a lack of IV fluid. What will you do when a critical patient comes in shock or hypoglycemic when you don’t have IV fluids or glucose at hand? They die in front of you from a disease that could have been easily averted in normal conditions.

[Female nurse]

*“There is no CBC [complete blood count] test. We could not know if a particular patient is having an infection or not. We are in the COVID-19 pandemic, but we do not have diagnostic tests or isolation sites for those who contract the disease. We see many healthcare professionals and patients showing symptoms of COVID-19.”* [Male resident physician].

### Lack of personal protective equipment

All participants mentioned that a lack of personal protective equipment was evident. The shortage of surgical and examination gloves, gauze, eye goggles, and face masks were the most frequently reported. Consequently, healthcare providers were required to wash disposable and surgical gloves for reuse, while others recalled that the patient would be requested to buy personal protective equipment. The participants described delivering newborns with bare hands, performing injections without gloves, and dressing wounds with unprotected hands, putting both themselves and patients at risk of infection.

*“I never imagined a day when we’d reuse gloves, hand-deliver without protection, and perform procedures not found in textbooks.”* [Male senior physician].

### Hazardous improvisation

Operating in cataclysmic circumstances led healthcare workers to develop many adaptation mechanisms, such as the use of expired medications for a wide range of diseases. This brought significant unease among the practitioners regarding the possible adverse effects of expired medicine over both the short and long term.

“*We are violating established medical guidelines. Using medications beyond the expiration date is unusual. The act of knowingly giving expired medicine can inflict significant psychological trauma and it is morally threatening.*” [Female Midwife].

The participants mentioned that due to the dire circumstances, they had to source gauze and cloth by requesting donations of used textiles from the community.

*“We are forced to go out and ask for traditional clothing from the community to utilize them as gauze.”* [Male nurse].

### Reduced number of staff and increased workload

There was almost complete agreement among the participants that the number of staff was significantly reduced following the siege. Five predominant reasons for the reduction in the number of care providers were: (1) Closure of ACSH, which led many residents and interns to return to their homes across Ethiopia; (2) Some care providers who had family members outside of Tigray left the region to join them because of the war; (3) Others joined their families due to food insecurity; (4) Some staff also sought jobs with NGOs to support themselves; (5) Some staff joined the Tigray military forces; and (6) Some staff members lost their lives due to the consequences of the war. Additionally, the number of care providers who left the hospital and whose whereabouts were unknown increased over time. The following quotes illustrate the shortages and consequences reported.

“We *[interns] were 180 in number before the siege. However, close to sixty of them have left, resulting in heavy workloads.”* [Female Intern].

*“Eight nurses from the emergency department have left the hospital and this has resulted in significant compromise on the service delivery.”* [Male Nurse].

“One *of our midwife colleagues passed away due to a lack of medication, while another lost his life to a gunshot wound as he sought refuge from the escalating hostilities that were happening in Mekelle.”.*” [Male Midwife]

It was also repeatedly mentioned that the reduced number of staff has resulted in a considerable additional workload for the staff remaining on duty. In addition to the reduced number of staff, the pattern analysis of the responses on specific reasons for the increase in workload showed the following: (1) there was an exponentially increasing number of patients related to the crisis in Tigray, (2) the primary and secondary healthcare levels were not functional due to collateral and targeted damage to the health facilities in the Tigray, increasing the number of patients in ACSH, and (3) absenteeism of healthcare staff was a serious problem due to the lack of fuel for transportation.

The reduced number of care providers and lack of fuel for transportation for care providers also resulted in longer working hours. The duration of working hours reported by the participants had increased from eight hours per day to 15 h and above per day, as described below.

‘We now work 36 hours for three days a day due to resident exodus following the siege.” [Female Resident physician].

“*As a resident physician, we were expected to read and work, but we are not reading and updating ourselves due to lack of internet service, [we are] too tired of the high workload and frequent duty turnover have negatively affected the academic quality and patient care.*” [Male Resident physician].

### Theme 2: personal survival threats posed by the siege

According to the study’s participants, the siege has had detrimental effects that have caused concern about the well-being of those providing care. Common survival concerns included food insecurity, drone attacks, and insufficient access to medications, both for themselves and their colleagues. Additionally, the participants noted that their own personal survival was at risk due to their inability to access their bank accounts and because they have been working without pay for the past nine months since the start of the siege.

### Hunger and food shortage: “eating a dinner has become questionable”

Healthcare workers have not received their monthly salary for nine months prior to the current study, and the banking system has been blocked. Moreover, they mentioned that they are facing severe food shortages.

*Many of us are now questioning whether we can even afford to have dinner, let alone support others. Due to not being paid our salaries and not being able to access our savings in the banks, we are struggling to support ourselves and those around us. It’s difficult to express the challenges we are facing in words* [Female, resident physician].

As per the participants, having even one meal a day had become a luxury. Most of them and their colleagues skipped breakfast and continued their work at the hospital without eating anything throughout the day. They also revealed that hunger had already started to pose a serious threat to the health professionals and their dependents. During the interview, one of the participants spoke in a slow and weak voice, with her eyes full of tears as she shared her story. *“… in our unit, because we spent the whole day up to 5 p.m., it has become customary that some colleagues who can afford bring food with them in the morning to share with us at the lunch time. On one of the days, we asked a colleague to join us for lunch. She said ‘no’! We ask why. [Her eyes full of tears], she said, ‘my kids were asking me to buy them bread in the morning. I did not buy them because I had no money to do so. I left my kids with nothing to eat at home. I left them for God to take care of them. I came here to work. How do you think I can eat?*” [Female Nurse].

Participants shared their observations from their workplace regarding the effects of hypoglycemia. They noticed that some of their colleagues would start sweating, while others would collapse while working, and some would even sleep during working hours due to hunger pains or cramps. Additionally, the participants witnessed some unusual scenarios in the hospital where some healthcare providers would search for food provided to patients by the hospital or among those who accompanied patients in cases where the patient refused to eat.

The region has been facing food insecurity due to the siege, forcing many to find ways to cope. Among the reported coping mechanisms are moving back in with parents to share food, sharing food with colleagues, and relying on hospital provisions. Some have resorted to seeking employment with humanitarian NGOs or joining armed groups to access food. Thus, participants have expressed that their lives and expectations have been greatly impacted by the siege, leading some to leave their hospital jobs in search of any paid work that could provide them with sustenance.

*“We do not have a salary […]. (Previously) we used to think about many things for the future. We used to think about owning cars etc. Now, we constantly think about what we are going to eat. We constantly ask ourselves if we have anything to be eaten. For a week, end to end, we think about what to eat. Everything is changed. Our life is totally changed. We stop thinking about the future, and then we start to think about our day-to-day lives.*” [Female Senior Physician].

### Risk of death: “my father has died due to a lack of medication.”

The participants strongly emphasized their concern about the looming risk of death to themselves and their family members as the siege has not ceased. Data analysis showed that much of the concerns related to the risk of death (both family members and themselves) as a result of drone attacks, jet bombardments, and lack of medication.

It was repeatedly mentioned that there is a high possibility of progression of the food shortage from hunger/food insecurity to the status of famine. A participant explained the looming risk of death due to famine in this way:

“*To me, I am seeing what concerns me is already happening. People are dying in front of us either due to hunger or lack of medication or any other disease related to it. I believe that the worst scenario has already started to happen.” [Female intern]*.

Participants witnessed the deaths of their colleagues and family members due to lack of medication, admission of their colleagues’ children to the hospital due to undernutrition, COVID-19 infection of colleagues and civilians suffering from the air strikes and drone attacks.

### Consequences related to physical well-being

The consequence of the siege has extended to the physical well-being of care providers. Tiredness and hypoglycemia, appetite loss, weight loss, lower back pain, and leg edema were the most frequently mentioned effects of the siege on the physical wellbeing of the healthcare providers. The participants related these negative consequences to food shortages and hunger, work and life-related psychological stress, excessive workload, and long working hours in the hospital. One participant described it this way:

“You *would see care providers hypoglycemic and sweating. They fell asleep while trying to assist patients to get up.*” [Female nurse].

Although an increasing number of complaints of lower back pain among care providers was stated, participants also repeatedly mentioned weight loss, facial aesthetic downgrading, and appetite loss for available food as negative consequences experienced regarding their physical wellbeing.

### Theme 3: social and other consequences of the siege among care providers

The lived experience regarding the negative health consequences of the siege emerged to affect the psychological, social, and physical well-being of care providers. The bi-directional interaction between the consequences was articulated by the participants.

### Consequences on psychological well-being

Work-related anxiety and dissatisfaction, life-related stress, hopelessness, sleeplessness, and lack of concentration at work were the most frequently reported manifestations experienced by caregivers.

While challenging work and living conditions during the siege took a toll on medical staff’s mental health, lack of supplies topped the list of anxieties. Interviewed providers expressed distress about failing patients due to limited drugs and equipment, knowing their skills could have helped if resources weren’t so scarce. This scarcity became a major source of psychological distress.

*“There is hopelessness, fatigue, and mental fatigue among us. You do not help patients who are in pain. You do not stop bleeding. You do not give patients fluids and glucose when they need it. This creates annoyance and hampers service delivery. It is difficult to work under such conditions. ” [Male Nurse]*.

Moreover, participants mentioned that the substandard care offered to patients due to lack of resources as the main source of anxiety and stress when they ended their shifts and went home. A nurse mentioned her feeling this way:

“*I have my own problems at home, but the challenges I face at work place are more devilish.*”

“*There is no oxygen, and you witness patients dying at their prime ages merely due to lack of oxygen. Oxygen interrupts frequently due to power outage. You get concerned whether the oxygen is available or not… whether the patient is alive or dead. When you go home, you don’t feel restful.” [Female Married Nurse]*.

### Life-related anxiety and stress: “… the sound of ‘Mama bread?’ is still ringing in my ears!”

Caregivers are experiencing stress and anxiety due to survival threats such as food insecurity, economic instability, and limited access to transportation and communication. Those with children are particularly impacted by these challenges. A nurse and a mother of young children explained it this way:

*“Sometimes I really question myself if I have a psychiatric illness. I don’t trust myself… I have left my kids at home. They were asking for bread. They were pulling my skirts! I couldn’t help. Still the sound: ‘mama bread! Mama bread!’ Is still ringing in my ears [cries for longer period]. It is only my flesh that is here. I am afraid I could harm patients. It is because I should be here, otherwise it is unbearable.” [Female nurse]*.

Another participant also described how the siege terminated her hope:

*“Due to the siege, my life has completely changed upside down. There were a lot of things I aspired to achieve at a personal level. They all vanish in to thin air. I am a mother; I couldn’t fulfill the basic requirements of my child. My husband lives in another part of Ethiopia, I have not heard of him so far. As there is no telecom or Internet service in the place where I am here. These pile up to the reasons why I should be anxious. I have significantly decreased my concentration at work”.* [Female Resident Physician]

### Sleeplessness

Sleep disturbances have become the new normal amongst health professionals.

*“When you go home, you roll-over in the bed the whole night. There are nights where I failed to fall asleep for a moment too. ” [Female Resident Physician]*.

Participants mentioned that the consequence of the siege are far reaching that they have developed new sorts of behavior.

*“Related to the war, there are new behavioral developments… we have developed sound hypersensitivity, we are conditioned to associated any loud sound, say the sound of the door shutting or opening, with airstrikes or heavy artillery shelling. Therefore, every family member has to react in a reflex way. You cannot sleep tight as used to be in the old times. ” [Male Senior Physician]*.

### Social isolation and loneliness: “If my pocket is empty, […] I would like to be alone.”

Participants reported that the siege resulted in a sense of social isolation and loneliness. Care providers were unable to provide socialization opportunities, and the cost of transportation prevented visits with family. Additionally, the communication blackout made it challenging to maintain contact with loved ones. The following quotations capture the above opinions.

“*If my pocket is empty, I would not be comfortable to join friends. I would like to be alone! You know why? Either they would expect me or I expect them [to cover the expenditure related to the refreshment cost]. That is why I prefer to be alone!*” [Male Resident].

*“Visiting family is almost impossible. The reason why is because there will be a problem related to the ongoing war and siege, which demands my involvement to deal with it. Hearing your family’s problem while you are unable to solve it adds pain to what already exists. Hence, I myself prefer to refrain from being socially connected.”* [Male Intern].

### Coping with psychological trauma

The participants mentioned multiple coping mechanisms for stress. Discussing the issue with colleagues, helping each other, reading books, visiting religious institutions, engaging at work, and entertainment were frequently mentioned. Most participants described that engaging in discussion to process the current condition would relieve their stress. Another participant also expressed his view as follows.

*“When I am done with my work, I tend to talk about the current situation with my friends. We discuss the siege, then we change themes of discussion. then we see tomorrow as if it will be bright. That is how we try to cope up with the stress.*” [Female Intern].

Other participants mentioned that visiting religious institutions relieves anxiety: *“I usually visit church. I address my prayer too. Or I weep at the church. Anyway, I get some relief there. The problem is that when I come home, I am back with problems altogether. You know, the current problem we are in is not easy, it will not go away simply. It is for a while that I try to stay engaged.*” [Female Resident physician].

The participants repeatedly mentioned staying engaged at work would relieve their stress.

*“I think staying engaged, and working your level best is an important escape mechanism. It is the best way to forget the stress of the siege. Knowing that you are contributing at least in some way will provide some relief. The fact is that it is not only because I am supporting patients, but I am also supporting myself. I will stay busy throughout the day. Thus, this will feed my brain and create some sort of satisfaction. The fact that I am helping will make me busy and I will also have mental satisfaction.*” [Male Senior].

### Theme 4: consequence of the siege on motivation and energy of health professionals

Despite a sustained decline in care provider availability due to the ongoing conflict, a contingent of personnel remained on duty, actively engaged in patient care nine months following the siege. A nurse stated this way:

*“Previously, people could not wait even a single day for their salary. But now look! Nine months have passed and people are still coming to work. This is a miracle for me! Despite their problem at home, such as hunger, they have continued to work.’’ [Married female nurse]*.

Health providers at ACSH are currently facing a decline in motivation and energy levels, largely attributed to the lack of medical supplies and personal protective equipment. This situation is causing healthcare workers to feel helpless and unable to provide the necessary care for their patients, ultimately leading to discouragement. Furthermore, it was discouraging that they had to direct patients to buy medication from private pharmacies despite knowing that these clients could not afford it. Patients suffering from kidney failure are currently without any viable treatment options. Several quotes related to this theme are presented below.

*“It really touches your morals. It makes you think of how we get to this hell. You feel anxious. You feel sad. Sometimes you get depressed when you think you could treat them. There are times I felt in deep grief. ’’ [Female Senior physician]*.

*“The existing drug shortage, the material shortages like gloves, decreases your motivation and energy. The healthcare worker becomes useless to the patient. We become like any layperson anywhere else. You just sympathize with the patient, and you send him home without any medication. When you see a patient in pain, you also become pained and you listen to your pain, helpless and hopeless with declined motivation.” [Male Nurse]*.

*“Seeing your sister, your brother, your mother, getting out of the hospital and not getting any treatment is very disturbing, that kills your motivation.” [ Male Resident physician]*.

The precariousness of life under siege, compounded by the months-long absence of their salary, further eroded the caregivers’ already dwindling motivation and energy. The following quotes capture the main source of demotivation to serve under siege;

*“The uncertainty of the situation is decreasing our motivation. We do not know what will happen tomorrow. Even if we come to work, we are not working with the hope and motivation we used to have.*” *[Female Resident Physician]*.

*“With the hunger, with the anxiety related to the siege, our motivation has seriously decreased. I do not think there is any staff working happily.” [Female nurse]*.

### Reason to be on duty “If there is something that I can offer, why should I not come?”

Despite not receiving their salaries, healthcare workers have tirelessly worked long hours, days, and weeks to serve their patients. When interviewed, these providers expressed that their professional obligations were a crucial factor in maintaining their motivation and energy at work, even in the midst of difficult circumstances. They cited their commitment to the Hippocratic oath they took at the start of their careers as a driving force behind their dedication to their work, even when unpaid.

A medical intern said, “*We joined medicine not for salary but to serve the dignified human being,”* while a male senior practitioner also said *“we vowed to serve the community; Humanity is the reason I have to go to work.”* Similarly, a resident said *“…there are patients more troubled than us in need of our help, which could mean death to the patients if we are not there to offer what we can”.*

Yet another clinician said this:

*“You should never give up! it is it! You must do what you are able to do. […] If there is something I can offer, why I should not come? Whether it is a care or psychological advice or what else, no matter. Whatever little it is, if you can contribute a fragment of care, coming and serving is mandatory.”* [Male Senior Practitioner].

Some respondents stated they believed that the siege as a component of the war being fought against their own racial or ethnic identity. Consequently, they felt motivated to protect and serve their community. They viewed it as an integral part of their struggle for survival as a particular race or ethnicity, or as a battle they were actively involved in. For example, a female nurse said: ‘… knowing that *someone in the battle is fighting to defend their people gives you the motivation and energy to continue serving patients”*.

Another mentioned “ As *Tigray people we are all on the battlefield. As a family, neighbors, and community, the suffering we are enduring is the same, and hence serving my people is the only better option left… In addition, as far as the siege is imposed on us because of our identity, we all need to be part of the solution at least by serving our community. […] Patients are part of our community just as your mother or your father…, How could someone say “ I’m not paid’ to treat a father of his own in a critical state? I believe that no one other than me must do it.”* [Female Intern].

A few participants reported that their hope for a return to normalcy drives them to fulfill their duty.

“You know *what you have been investing in for a lifetime shouldn’t be left aside simply. There could be challenges, but you will never cut your role down. Hopefully better days will come.”* [Male Nurse].

## Discussion

This study aimed to bring to light the impact of the Tigray siege on healthcare service delivery by exploring the lived experiences of healthcare workers. The present study revealed that healthcare workers are routinely concerned about the risk of death themselves, for their relatives, and their patients due to hunger, air bombardment/drone strike, and lack of essential drugs and essential services like transportation and telecommunications. Study participants indicated that concerns about food insecurity among healthcare professionals stem from the fact that staff have been unpaid for the last 9 months, with no access to banking, and a high cost of living related to the de facto blockade.

Essential medical supplies, equipment, and personal protective materials were largely unavailable during interviews, hampering essential healthcare services. This left health professionals anxious and devastated as they did not provide appropriate care. It is well recognized in the literature that a lack of medical supplies impedes essential healthcare services [10, 29]. The present study has also revealed that despite the dire circumstances, health professionals are devising desperate but hazardous improvisation of care. Treatment of both communicable and non-communicable diseases, and performing elective and emergency surgery have become impossible resulting in a huge death toll. Many war-torn and besieged areas in the world such as Syria have sustained the breakdown of the chain of medical supplies, resulting in significant loss of life [11]. The prolonged siege of Tigray, which lasted for nine months, has led to an unprecedented humanitarian crisis. More than seven million people have been suddenly cut off from essential medical supplies, including hospitals. Moreover, humanitarian agencies have been unable to access the besieged population. In comparison to the Syrian study where the UN reported that 400,000 were under siege, the present study shows a larger population affected and a longer duration of the siege [11, 30, 31].

Health professionals face many difficulties in treating patients, such as making a diagnosis without laboratory tests and imaging modalities. Procedures are being performed without essential diagnostic tests. Furthermore, expired drugs were utilized out of desperation. Misdiagnosis and subsequent mismanagement of patients lead to significant increases in morbidity and mortality. As 70–80% of the health facilities in Tigray have been ransacked and/or destroyed, ACSH is the only institution where people can get service. However, the Hospital is far from providing standard healthcare service [12, 18]. Compared to studies on health services in war zones, this study provides the first qualitative report on hazardous improvisations including the utilization of expired drugs and the re-use of surgical gloves in dire circumstances [12, 13, 15].

The war and subsequent siege have resulted in significant staff attrition and turnover. Health professionals have left the hospital for food insecurity reasons, while others joined the Tigray military forces and nongovernmental offices in a quest for survival. This has also been reported in war-ridden areas elsewhere [11, 12, 32, 33]. In violation of the Geneva Conventions, health professionals have been targeted by combatants during the war [26]. Deaths of health professionals due to both direct and indirect effects of the war have negatively affected the number of staff. Moreover, there are health professionals whose whereabouts are unknown. The remaining staff are suffering from workload as a result. This has become a paramount issue in the physical and psychological health of the providers. Inadequate staffing coupled with deterioration in the health of clinicians is another hindrance to providing optimal care [18, 34]. An additional contributing factor to the poor quality of healthcare services at ACSH could be the underlying shortage of medical personnel. This shortage is further exacerbated by the ongoing conflict in Tigray, which has led to the displacement of many healthcare workers. As a result, ACSH is understaffed and unable to provide adequate care to all patients [18, 20, 21].

The hospital encountered various obstacles that have impacted its functioning. Chief among these is the absence of fundamental amenities, including transportation, telecommunication, electricity, and banking systems. Specifically, the absence of transportation services has led to health professionals arriving late or missing shifts, resulting in compromised patient care and preventable fatalities. Additionally, frequent power outages hindered medical care at the hospital. Essential equipment, such as mechanical ventilation and the oxygen plant, malfunction during these outages, leading to unexpected loss of life [35]. This study delved into the distinct challenges presented by conflict in areas where even fundamental amenities, such as electricity, communication, transportation, and banking, are entirely absent. In contrast to past research, which has examined settings with varying degrees of infrastructure, the present study shed light on the unique difficulties posed by conflict in a setup with no functioning infrastructure [12, 13, 33].

It is observed in this study that the right to health which is stated in the general comment No.14 of the United Nations Economic and Social Council, as non-dirigible as it might be, has been violated as the healthcare workers included in our study stressed their survival concerns related to food insecurity, violent attack and, lack of essential medicines [27]. Congruent to the concerns of participants in this study, a report has shown that the population in need of emergency food assistance in the Tigray region has reached 5.2 million. Due to the siege, the Tigray region of northern Ethiopia stands in a state of humanitarian disaster. This has affected the general population and particularly those who provide healthcare for the population; seeing nurses and doctors queuing for humanitarian food parcels has become a new normal [36, 37].

The main drivers of psychological disarray of the health professionals in this study were working with limited supplies and equipment, increased workload, absent salaries, and food security. Health workers were forced to improvise with hazardous tools to continue providing their services. Another important source of psychological burden leading to anxiety, stress, depression, and sleeplessness was the health professionals’ inability to support themselves and their family members. Similar to other healthcare workers living in war-torn areas, these psychiatric manifestations not only affect the care providers but also interfere with their ability to support others [37]. In a study conducted in Iraq states that the main source of psychological burden is mainly due to the impact of handling mass casualties [38]. In the present study, the burden is dual, handling mass causalities, and security and economic threats of loved ones. To this end, health professionals in the present study are at highest risk of burnout, compassion fatigue, and vicarious trauma [39–41].

Strategies for coping with the siege included discussion with colleagues, reading books, and participating in work. Psychological strategies such as visiting religious institutions and prayers were also other coping mechanisms. Similar coping mechanisms were mentioned amongst health professionals of Uganda, Yemen, and Syria. Similarly, health personnel showed considerable resilience in other conflict areas as well [11–13, 32, 33, 42–45]. The peculiar challenge to these coping mechanisms among the participants of the current study would be that those religious institutions might not be safe at all. There were reports of systemic targeting of religious institutions during the Tigray war [46].

The motivation to work has declined among healthcare workers due to various reasons, including inadequate salary and conflicts between personal and professional responsibilities. Frequently, medical professionals encounter a scarcity of medication and equipment despite their presence at the hospital. This can prove to be a significant hindrance for healthcare workers as they strive to offer the required attention, leaving them with a sense of inadequacy in the face of their patients’ distress. Previous studies have indicated that such episodes can lead to a decline in morale among healthcare workers [13, 43, 45]. The factors identified in this study have also been recognized in the studies done in Uganda and Syria [13, 43]. In the Ugandan study, a good working environment with adequate medical supplies plus regular and adequate pay has been described as a crucial motivator to work [13]. In the current study, the healthcare system had completely collapsed, leaving no institution capable of paying even the basic salaries for healthcare professionals. The study motivators used in Uganda could not be applied in the current study [45].

Despite not receiving their salary, healthcare workers have continued to work with professionalism and ethical standards. The pledge that a healthcare professional takes at the beginning of their career appears to be a significant motivating factor for them to continue working even in difficult circumstances. This has been observed in similar studies conducted in Uganda and Syria [13, 43, 47]. Another motivator for healthcare workers to persist in their work is the ongoing battle against a particular ethnic group and region, where external entities are alleged to be attempting to eradicate that population. These healthcare workers perceive their efforts as a crucial part of the fight against extinction, viewing it as a fight for survival. This study’s findings diverge from those of a Ugandan study, which identified leadership engagement, appreciation, and community support as significant motivators. This suggests that motivational factors may vary between different contexts or populations [13].

## Conclusion

The current study illustrated that the siege on Tigray has severely and irrevocably affected the lives of healthcare providers at the ACSH, Tigray, Ethiopia. The siege posed a double burden to healthcare providers both because of the blockage of basic infrastructure and humanitarian services, as well as to compromised care to meet the demands of an increasingly desperate population of patients. These conditions created survival concerns with an attendant high risk of mental, social, and physical health consequences. The current study strongly recommends that warring parties execute global, regional, and local laws aimed at ensuring access unfettered humanitarian services including inputs for food and healthcare provision in war-torn areas. Furthermore, the economic, psychological, and social needs of healthcare professionals during war and post-war period should be addressed.

### Electronic supplementary material

Below is the link to the electronic supplementary material.

Supplementary Material 1

## Data Availability

The datasets generated and/or analyzed during the current study are not publicly available but are available from the corresponding author at a reasonable request.

## Abbreviations

ACSH: Ayder Comprehensive Specialized Hospital
CBC: Complete blood count
OR: Operation theater
NGO: Non-governmental organization

## References

[1] Stewart F, Holdstock D, Jarquin A (2002). Root causes of violent conflict in developing countries Commentary: conflict—from causes to prevention?. BMJ.

[2] Sidel VW, Levy BS. The health impact of war. Int J Inj Contr Saf Promot. 2008;15(4):189– 95. doi: 10.1080/17457300802404935. PMID: 19051082. 10.1080/17457300802404935 19051082

[3] Summerfield D. War and mental health: a brief overview BMJ 2000; 321:232 10.1136/bmj.321.7255.232. 10.1136/bmj.321.7255.232 PMC1118225 10903662

[4] Harrijvan M, Weerdesteijn M (2020). To appease or to repress: how dictators use economic dynamics to increase their regime longevity. Crime Law Soc Change.

[5] Mehmet, Gurses, Mason TD (2010). Weak States, Regime types, and Civil War. Civil Wars.

[6] Card C (1996). Rape as a Weapon of War. Hypatia.

[7] Kirby P (2013). How is rape a weapon of war? Feminist International relations, modes of critical explanation and the study of wartime sexual violence. Eur J Int Relat.

[8] Eunice Hammond. The Effects of War On Healthcare Infrastructure And Health Workers: Chaos Compounding Death and Injury. 2018. https://theowp.org/the-effects-of-war-on-healthcare-infrastructure-and-health-workers-chaos-compounding-death-and-injury/.

[9] Blyth DM, Yun HC, Tribble DR, Murray CK (2015). Lessons of war: Combat-related injury infections during the Vietnam War and Operation Iraqi and Enduring Freedom. J Trauma Acute Care Surg.

[10] Ameh C, Bishop S, Kongnyuy E, Grady K, Van den Broek N (2009). Challenges to the Provision of Emergency Obstetric Care in Iraq. Matern Child Health J.

[11] Ekzayez A, Alhaj Ahmad Y, Alhaleb H, Checchi F (2021). The impact of armed conflict on utilisation of health services in north-west Syria: an observational study. Confl Health.

[12] Elnakib S, Elaraby S, Othman F, BaSaleem H, Abdulghani AlShawafi NA, Saleh Al-Gawfi IA, Shafique F, Al-Kubati E, Rafique N, Tappis H (2021). Providing care under extreme adversity: the impact of the Yemen conflict on the personal and professional lives of health workers. Soc Sci Med.

[13] Justine Namakula S, Witter, Living through conflict and post-conflict: experiences of health workers in northern Uganda and lessons for people-centred health systems, Health Policy and Planning, Volume 29, Issue, suppl_2 S. 2014, Pages ii6–ii14, 10.1093/heapol/czu022. 10.1093/heapol/czu022 PMC4202915 25274642

[14] Murthy RS, Lakshminarayana R (2006). Mental health consequences of war: a brief review of research findings. World Psychiatry.

[15] Sabes-Figuera R, McCrone P, Bogic M, Ajdukovic D, Franciskovic T, Colombini N (2012). Long-term impact of War on Healthcare costs: an eight-country study. PLoS ONE.

[16] https://www.icn.ch/news/reports-more-1200-incidents-violence-against-health-care-2019-demand-accountability.

[17] The Guardian Editorial. The Guardian view on healthcare in war: Protect those who aid others. 2019. https://www.theguardian.com/commentisfree/2022/feb/15/the-guardian-view-on-healthcare-in-war-protect-those-who-aid-others.

[18] Gesesew H, Berhane K, Siraj ES (2021). The impact of war on the health system of the Tigray region in Ethiopia: an assessment. BMJ Global Health.

[19] Dyer O (2022). Tigray’s hospitals lack necessities as relief supplies are blocked. say Doctors BMJ.

[20] Clarfield AM, Gill G, Leuner CJ, Moses AE, Paltiel O (2022). An appeal to world leaders: restore health care for ethiopians in Tigray. Lancet.

[21] Paltiel O. A M. Clarfield 2022 Tigray: the challenges of providing care in unimaginable conditions. BMJ 376 o400 10.1136/bmj.o400. 10.1136/bmj.o400 35168941

[22] Kherallah M, Alahfez T, Sahloul Z, Eddin KD, Jamil G (2012). Health care in Syria before and during the crisis. Avicenna J Med.

[23] Rawaf S, Hassounah S, Dubois E (2014). Living conditions in Iraq: 10 years after the US-led invasion. J R Soc Med.

[24] Physicians for Human Rights. Medical personnel are targeted in Syria. 2020 [cited 9 Feb 2020]. https://phr.org/our-work/resources/medical-personnel-are-targeted-in-syria/.

[25] ICRC. Even wars have limits: Health-care workers and facilities must be protected. 2016. https://www.icrc.org/en/document/hcid-statement.

[26] The Geneva Convention. Hospital (Lond 1886). 1899;27(683):68. PMID: 29838474; PMCID: PMC5269171. https://www.ncbi.nlm.nih.gov/pmc/articles/PMC5269171/. PMC5269171 29838474

[27] Security Council. Security Council Adopts Resolution 2286. (2016), strongly condemning attacks against medical facilities, personnel in conflict situations| Meetings Coverage and Press Releases. United Nations. 2016).

[28] Koteiche R, Murad S, Heisler M. My only crime was that I was a doctor. Physicians Hum Rights. 2019. https://phr.org/our-work/resources/my-only-crime-was-that-i-was-a-doctor/#phr_toc_0)).

[29] Berhe E, Paltiel O, Gebrearegay H, Abraha HE, Tequare MH, Teka H, Mulugeta A (2022). Ethiopia’s Tigray dialysis service cut due to dwindling supplies amid war. Kidney Int Rep.

[30] Report of the Secretary-General on the implementation of Security Council resolutions 2139. (2014), 2165 (2014), 2191 (2014), 2258 (2015), and 2332 (2016) concerning the Syrian Arab Republic. https://digitallibrary.un.org/record/817581.

[31] Doctors say lives. are lost in hospitals in Ethiopia’s Tigray due to dwindling supplies, blame blockade. Reuters. https://www.reuters.com/business/healthcare-pharmaceuticals/doctors-say-lives-are-lost-hospitals-ethiopias-tigray-due-dwindling-supplies-2022-0%E2%80%A6.

[32] Ajdukovic M, Ajdukovic D (1998). Mental health care for helpers: experiences from a training programme. Libby Tata Arcel, War violence, trauma and the coping process.

[33] Witter S, Wurie H, Chandiwana P, Namakula J, So S, Alonso-Garbayo A, Ssengooba F, Raven J. How do health workers experience and cope with shocks? Learning from four fragile and conflict-affected health systems in Uganda, Sierra Leone, Zimbabwe and Cambodia. Health Policy Plan. 2017;32(suppl_3):iii3-iii13. 10.1093/heapol/czx112. PMID: 29149313. 10.1093/heapol/czx112 29149313

[34] ICRC, Protocol I. (51) Protection of the civilian population. In: Protocol Additional to the Geneva Conventions of 12 August 1949, and relating to the Protection of Victims of International Armed Conflicts (Protocol I), Entry into force 7 December 1979. 1977.

[35] Debarre A. Hard to Reach: Providing Healthcare in Armed Conflict. International Peace Institute; December 2018.

[36] Ethiopia civil war.: Doctors among those begging for food in Tigray [Internet]. BBC News. 2022 [cited 2 February 2022]. Available from: https://www.bbc.co.uk/news/world-africa-60169326?at_medium=RSS&at_campaign=KARANGA

[37] Warning over fuel and food stocks as. ‘hellish’ Tigray reels from airstrikes [Internet]. the Guardian. 2022 [cited 2 February 2022]. Available from: https://www.theguardian.com/global-development/2022/jan/13/warning-fuel-and-food-stocks-hellish-tigray-reels-from-airstrikes.

[38] Faraj AA, Perez RL, Abbas AK (2014). The psychological impact of war on health professionals; a preliminary study. SL J Psychiatry.

[39] Berhe E, Tesfay B, Teka H (2022). Vicarious trauma on the hemodialysis healthcare workers in the besieged Ethiopia’s Tigray region: a call to action. BMC Med.

[40] Forrest L, Abdurrahman M, Ritsma A, Hategan A, Saperson K, Harms S, Waters H (2020). Recognizing Compassion fatigue, vicarious trauma, and Burnout. Humanism and resilience in Residency Training.

[41] Owen RP, Wanzer L (2014). Compassion fatigue in military healthcare teams. Arch Psychiatr Nurs.

[42] Joy Turyahabwa1, Ojiambo Ochieng R2, Were-Ogutte J. Gayfor V3, Howard Diawara L4, Eugene Kinyanda5 Impact of Conflict on The Psychological Wellbeing Of Health Workers In Liberia.

[43] Kallström A, Al-Abdulla O, Parkki J (2022). I don’t leave my people; they need me: qualitative research of local health care professionals’ working motivations in Syria. Confl Health.

[44] Bird K. Using life history research as part of a mixed methods strategy to explore resilience in conflict and post conflict settings, 2008 http://www.chronicpoverty.org/uploads/publication_files/bird_mixed_methods.pdf.

[45] Namakula J, Witter S, Ssengooba F, Ssali S. Health workers’ career paths, livelihoods and coping strategies in conflict and post-conflict northern Uganda. A Research Report; 2013.

[46] Pellet P. (2021). Background, causes and consequences of the Tigray conflict in Ethiopia that started on November 4, 2020. 28.

[47] Miles SH (2004). The hippocratic oath and the Ethics of Medicine.

