# Supplementary material for: Lived experience of healthcare providers amidst war and siege: a phenomenological study of Ayder Comprehensive Specialized Hospital of Tigray, Northern Ethiopia
Source: BMC Health Serv Res. 2024 Mar 6;24:292. doi: 10.1186/s12913-024-10655-3 (PMC10916075; doi:10.1186/s12913-024-10655-3)
Supplement: Supplementary file 1 — Supplementary Material 1 [file 12913_2024_10655_MOESM1_ESM.docx]

Interview guide

Thank you for your willingness to take part in this study and for granting permission to record audio. My name is ________________________, I am a member of the study team conducting a qualitative study on living conditions at Ayder Comprehensive Specialized Hospital staff. I sincerely appreciate your willingness to share your experiences regarding the general health condition, life, and work of health professionals at Ayder Comprehensive Specialized Hospital during times of war and siege. I kindly request that you provide me with a detailed description to the best of your ability. You can stop or withdraw from the interview at any time. The qualitative interview will be used solely for study purposes and confidentiality and privacy will be maintained. The interview will cover events that have taken place between June 28, 2021 and today.

**Socio-demographic characteristics of participants**

Date of Interview:

Interview Start Time: ____________End time: _____________

Name of Interviewer:

Name of Supervisors:

| Participant’s code | Age | Sex | Educational status | Marital status | Profession | Role in the department (if any) |
| --- | --- | --- | --- | --- | --- | --- |
| P1 |  |  |  |  |  |  |

**Man Questions**

| **Main question** | **Probing questions** |
| --- | --- |
| 1. Would you tell me about your professional background, including your role, and years of experience? |  |
| 2. Can you tell me about your personal experiences working at Ayder Comprehensive Specialized Hospital during the war and siege, including any challenges or difficulties you faced? |  |
| 3. Starting from June 28, 2021, how effect do you think the ongoing siege and war posed on professionals work hours? Would you tell me your personal experience? Additionally, how does this compare to your work hours before the war? | Probe: Please elaborate on your perception of shifts, staff punctuality, and shift rotations. |
| 4. Has there been any change in the hospital task force? How many staff members are currently working, and how many have left Tigray? | - Can you provide me the reason why they have left the hospital - Have there been any changes in working hours as a result? How does this affect the quality of care provided? |
| 5. How has the war and siege affected the overall functioning of Ayder Comprehensive Specialized Hospital? | - Effect on provision of health services at Ayder Comprehensive Specialized Hospital? - In reference to your unit/department, how do you evaluate the effect on adequacy of medical supply and equipment - Have there been any changes or improvisations in service provision or treatment protocols? Please provide examples |
| 6. Have there been any changes in your role as a health professional due to the war and siege? If so, please provide personal examples. |  |
| 7. In your opinion, how has this war and siege affected the living conditions of health professionals at Ayder Comprehensive Specialized Hospital? | - Please explain how it has affected them and share any personal encounters you may have had. - General Health, mental health manifestations, social interaction, appetite, sleep pattern |
| 8. Have there been any changes in your own life due to this war and siege? Please explain | - Can you discuss any personal survival threat posed by the war and siege - Can you discuss any physical and mental health issues that you encountered as a result of working during war and siege? Please provide examples - Have your family members been directly affected by this war and siege? Please explain |
| 9. Personally, how has the war affected your morale and energy levels? Please explain in detail and provide specific examples. | - Motivation, Job satisfaction, Change in commitment - How would you assess the morale and energy levels of your colleagues? |
| 10. Would you tell me the effct of war on staffs stress or anxiety? effects that you feel Are you stressed? If so what are your main stressors during this time? | - What makes you feel like that? What personal feelings do you experience in reference to the siege and war? - How do you cope with stress? What coping mechanisms have proven effective for you? |
| Despite not being paid for eight months and facing problems due to the ongoing siege, what motivates you to continue showing up for work? Explain | - What are the main fears or concerns that arise from this war and siege situation? |
| Finally, I will give you an opportunity to share any additional thoughts or experiences related to being a health care provider during war and siege. | - Once again, thank you for taking the time to participate in this interview. |
|  |  |
